# Non-invasive endothelial function assessment in patients with neurofibromatosis type 1: a cross-sectional study

**DOI:** 10.1186/1471-2261-13-18

**Published:** 2013-03-11

**Authors:** Luiza O Rodrigues, Luiz Oswaldo C Rodrigues, Luisa Lima Castro, Nilton A Rezende, Antonio Luiz P Ribeiro

**Affiliations:** 1 Department of Internal Medicine, Federal University of Minas Gerais, Av. Prof. Alfredo Balena, 190-246, Belo Horizonte, MG, Cep:30130-100, Brazil

**Keywords:** Neurofibromatosis type 1, Endothelial dysfunction, Peripheral arterial tonometry, Reactive hyperemia

## Abstract

**Background:**

Neurofibromatosis type 1 (NF1) is a multi-systemic disease caused by neurofibromin deficiency. The reduced life expectancy of patients with NF1 has been attributed to NF1-associated malignant neoplasms. However, an analysis of death certificates in the USA suggests that vascular disease could be an important cause of early death among these patients. Endothelial dysfunction (ED) is related to vasculopathy and is an early marker of subclinical atherosclerosis. Since neurofibromin has already been demonstrated to affect endothelial cell function, ED may be associated with NF1. The purpose of this study was to assess endothelial function in patients with NF1 using a non-invasive method.

**Methods:**

NF1 patients and healthy control subjects, aged 18 to 35 years, were included. Subjects were excluded if they had any risk factor for vascular disease or any other condition known to affect endothelial function. Endothelial function was assessed using reactive hyperemia-peripheral arterial tone (RH-PAT) technology. ED was defined as a reactive hyperemia index (RHI) lower than 1.35.

**Results:**

Four of the 29 (13.8%) NF1 patients and 1 of the 30 (3.3%) healthy volunteers had ED (*p* = 0.153). RHI medians and interquartile intervals were 1.8 (1.58-2.43) for the NF1 group and 2.02 (1.74 – 2.49) for the control group (p = 0.361).

**Conclusion:**

The prevalence of ED was similar in NF1 patients and healthy controls.

## Background

Neurofibromatosis type 1 (NF1) is an autosomal dominant genetic disease caused by mutations in the neurofibromin-encoding gene, located on chromosome 17q11.2. Neurofibromin is a tumor suppressor protein and NF1 is one of the most common monogenetic diseases, with a prevalence of 1:3,500 to 5,000 [1].

NF1 is a multi-systemic disease that presents with complete penetrance, but an extremely variable expressivity. The most common clinical features are multiple café-au-lait spots, dermal and plexiform neurofibromas, axillary and/or inguinal freckling, Lisch nodes, and cognitive and muscle-skeletal disorders [2]. NF1 has a large impact on patient quality of life, because of its associated morbidities, its aesthetic complications, its unpredictable chronic clinical course, and the possibility of affecting descendants.

NF1 has been shown to reduce life expectancy by approximately 15 years [3,4], a reduction attributed to NF1-associated malignant neoplasms. Surprisingly, however, a population-based analysis of death certificates in the USA [3] suggested that vascular disease may be an important cause of death in younger patients in NF1. In contrast, a similar study performed 10 years later in an Italian population [4] found that the rate of vascular disease-associated deaths in NF1 patients was lower. Despite this difference and the limitations of death certificate analyses, these findings were of interest, because they suggested that vascular disease, a potentially treatable or preventable complication, might be a main cause of early death in individuals with NF1.

The pathogenesis of NF1 vasculopathy is not well understood [5]. Neurofibromin expression has been demonstrated in endothelial and smooth muscle vascular cells in murine models of NF1 [6,7]. Chronic vascular inflammation observed in humans with NF1 and in murine models of NF1 [8] may be a cause of endothelial dysfunction (ED).

ED is an early event in the development of atherosclerosis. Several risk factors can lead to endothelial activation, which induces the expression of genes that are normally suppressed [9]. Changes in vascular morphology are preceded by ED, which also contributes to atherosclerotic plaque growth and clinical vascular disease [10].

ED is a systemic process, and there is an established correlation between the coronary and the peripheral vasculature allowing the development of non-invasive techniques to assess endothelial function. EndoPAT (Itamar Medical, Israel) equipment is a non-examiner dependent, non-invasive technology known as “reactive hyperemia peripheral arterial tone” (RH-PAT) technology. This system detects pulsatile plethysmographic signals through external finger probes, which are then digitally recorded and analyzed with the equipment’s software. This technique, also called PAT, has been validated for the assessment of endothelial function in patients with peripheral and coronary atherosclerotic disease (CAD) [11,12].

The present study aimed to non-invasively assess endothelial function in young adult NF1 patients using the PAT technology.

## Methods

This study was designed as a cross-sectional analysis to measure the difference in the prevalence of ED between a group of patients with NF1 and a group of participants without NF1. The other characteristics of the groups were similar. The institution’s ethics committee approved the study.

The sample of participants in the NF1 group was non-probabilistic and was selected from medical files at the Neurofibromatosis Outpatient Reference Center (NORC), by age (ranging from 18 to 35 years old) and place of residence (to ensure compliance with protocol visits). The estimated necessary sample size to find a 30% between group difference in the percentage of subjects with ED, with a power of 80% and an alpha error of 5%, was calculated to be 27 (results from OpenEpi, Version 2, open source calculator, sample size for cross-sectional studies).

Patients with NF1 aged 18 to 35 years old and residing in Belo Horizonte (MG - Brazil) were selected from their NORC files. All patients were diagnosed clinically, based on the presence of two or more of the following NIH consensus criteria: six or more café-au-lait spots, 2 or more ordinary neurofibromas or 1 plexiform neurofibroma, axillary and/or inguinal freckling, optic glioma, 2 or more Lisch nodules, osseous dysplasia of the sphenoid wing or tibia, and a first degree relative with NF1. To avoid the inclusion of patients with Legius syndrome [13,14], patients who presented with café-au-lait spots and intertriginous freckling had to also have a third diagnostic criterion for NF1. Patients were contacted by telephone. Healthy volunteers were recruited from the area located around the Federal University of Minas Gerais by personal or e-mail communication.

NF1 patients were included if they had been diagnosed with NF1, were aged between 18 and 35 years old and provided written informed consent. Control volunteers were included if they were aged between 18 and 35 years old and provided written informed consent. Both NF1 patients and control volunteers were excluded if they had any risk factor for CAD, including arterial hypertension, obesity, family history of early CAD or unknown, dyslipidemia, diabetes mellitus or glucose intolerance and smoking; or used any medication affecting endothelial function, including nitrates, alpha- and beta-blockers, calcium channel blockers, angiotensin converting enzyme inhibitors and statins, less than 48 hours prior to examination.

The height and weight of all were measured; and level of physical activity, smoking habits and the use of hormonal contraceptives were self-reported. Active individuals were defined as those who engaged in more than 1 hour per week of self-defined moderate to high intensity physical activity. Smokers were defined as individuals who had smoked tobacco during the last month. All smokers were excluded from the analysis.

Endothelial function was tested using EndoPAT (Itamar Medical). Each participant assumed a dorsal decubitus position with both arms resting relaxed on supports, in a silent and temperature-controlled (21° to 24°C) environment with low-light, according to the manufacturer’s instructions. Probes were placed on the second fingers of both hands. Signals were acquired for 15 minutes, with the first 5 minutes considered baseline, the next 5 minutes considered the arterial occlusion period, and the last 5 minutes considered the reactive hyperemia period. Arterial occlusion was achieved using an aneroid cuff inflated to 200 mmHg of pressure on the non-dominant arm.

All volunteers fasted for 3 to 12 hours before the test. Blood samples were collected and tests of thyroid function (TSH, FT4), lipid profile (cholesterol and triglycerides), fasting blood glucose, blood count (RBC, WBC and platelets), liver profile (AST, ALT, bilirubin, prothrombin activity), and kidney function (serum urea, serum creatinine) were performed. Subjects whose test results were not within normal ranges were excluded from the analysis.

**Figure 1:**
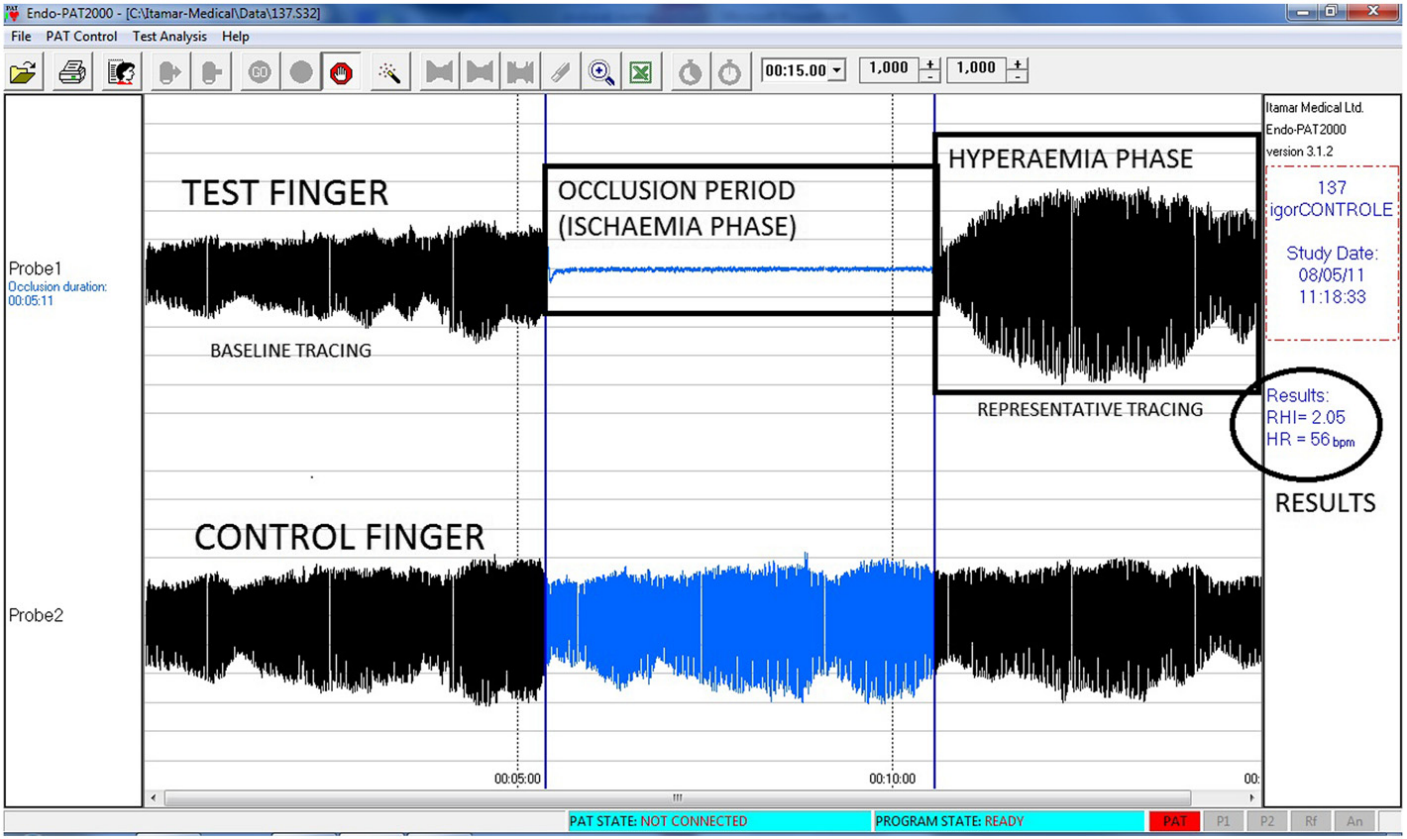
**Automatic analysis screen of the computer software, showing test results in one participant.** Abbreviations: RHI: reactive hyperemia index. HR: heart rate. Source: Itamar Medical Ltd. Software Version 3.2.4.

The dependent variable was the reactive hyperemia index (RHI), which is automatically calculated by the EndoPAT software (Figure 1) as the ratio of the average amplitude of the PAT signal over a 1-minute time interval, starting 1 minute after cuff deflation, divided by the average amplitude of the PAT signal over a 3.5-minute time period before cuff inflation (baseline). PAT index values from the study arm were normalized to those of the control arm, yielding the RHI final result [12]. RHI is not a normally distributed variable; therefore, it was analyzed with non-parametric statistical tests. RHI was transformed into its natural logarithm (LnRHI), which is normally distributed, for further analysis with parametric tests. The significance level was set at 0.05 (p < 0.05).

Independent variables included age, proportion of women to men, proportion of active to sedentary individuals, weight, height, body mass index, arterial blood pressure and resting heart rate.

Since co-variables differently distributed in the two groups could influence RHI results, a covariance analysis was performed to adjust for this possibility. All statistical analyses were performed with IBM SPSS Statistics software, version 18.

## Results

Twenty-nine NF1 patients and 30 healthy volunteers were evaluated. Their baseline demographic characteristics were similar, including age, ratio of women to men, ratio of physically active to inactive participants, body weight, body mass index, and resting arterial systolic and diastolic blood pressure. Mean height was significantly lower and mean resting heart rate was significantly higher in the NF1 than in the control group, and mean body mass index was significantly higher for women in the NF1 than in the control group (p < 0.05 each). Group characteristics are summarized in Table 1.

**Table 1:** Characteristics of the participants in the NF1 and control groups

	**NF1 (n = 29)**	**Control (n = 30)**	***P***
Age (years)	25 ± 4	24 ± 3	0.219
Women	23(79%)	21(70%)	0.412
Physically active	7(24%)	14(46%)	0.071
Weight of women (kg)	55 ± 9	56 ± 6	0.692
Weight of men (kg)	69 ± 8	73 ± 6	0.303
Stature of women (cm)	158 ± 6	164 ± 6	**0.003**
Stature of men (cm)	173 ± 5	179 ± 4	**0.038**
Body mass index of women (kg/m^2^)	22,0 ± 2,4	20,8 ± 1,5	**0.046**
Body mass index of men (kg/m^2^)	22,9 ± 2	22,7 ± 1,3	0.812
Systolic blood pressure (mmHg)	113 ± 9	114 ± 7	0.974
Diastolic blood pressure (mmHg)	72 ± 7	75 ± 8	0.300
Resting heart rate (bpm)	76 ± 7	69 ± 8	**0.002**

Data shown as means ± standard deviations or absolute count (percentage).

Blood test results were within normal ranges in all participants (Table 2). Some mean values showed significant between group differences, but these differences were not clinically relevant.

**Table 2:** Blood test results of the participants in the NF1 and control groups

**Blood test**	**NF1**	**Controls**	**P**
Total cholesterol – mg/dL	168 ± 35	182 ± 36	0.137
LDL cholesterol – mg/dL	99 ± 28	107 ± 30	0.309
HDL cholesterol – mg/dL	53 ± 12	58 ± 13	0.169
VLDL cholesterol – mg/dL	15 ± 7	17 ± 6	0.369
Triglycerides – mg/dL	78 ± 36	85 ± 34	0.452
Fasting glycaemia – mg/dL (70–99)*	83 ± 5	81 ± 5	0.169
Serum creatinine – mg/dL (0.4-1.3)*	0.66 ± 0.13	0.77 ± 0.14	**0.005**
Serum urea – mg/dL (15–40)*	27 ± 8	26 ± 7	0.768
AST - U/L (4 – 38)*	21 ± 6	26 ± 5	**0.001**
ALT - U/L (4 – 38)*	20 ± 19	20 ± 9	0.907
Serum total bilirubin – mg/dL (up to 1.2)*	0.75 ± 0.52	0.75 ± 0.35	0.986
Direct bilirubin – mg/dL (up to 0.4)*	0.28 ± 0.10	0.30 ± 0.11	0.432
Indirect bilirubin – mg/dL	0.49 ± 0.48	0.45 ± 0.28	0.703
Prothrombin activity - % (70–100)*	84 ± 13	90 ± 7	**0.034**
TSH - mcIU/mL (0.44 – 4.48)*	1.77 ± 0.89	1.67 ± 0.76	0.649
Free T4 - ng/dL (0.8 – 1.9)*	1.29 ± 0.17	1.16 ± 0.16	**0.006**
Hemoglobin – g/dL (12–18)*	14.1 ± 1.2	14.4 ± 1.1	0.298
Hematocrit - % (35–52)*	41.5 ± 3.4	42.0 ± 2.9	0.499
WBC count -/mm^3^ (4,000-11,000)*	6,933 ± 1,924	5,798 ± 1,438	**0.013**
Platelet count -/mm^3^ (140,000-450,000)*	262,758 ± 61,586	250,620 ± 62,143	0.458

Data shown as means ± standard deviations.

* Normal reference range of the test.

**Figure 2:**
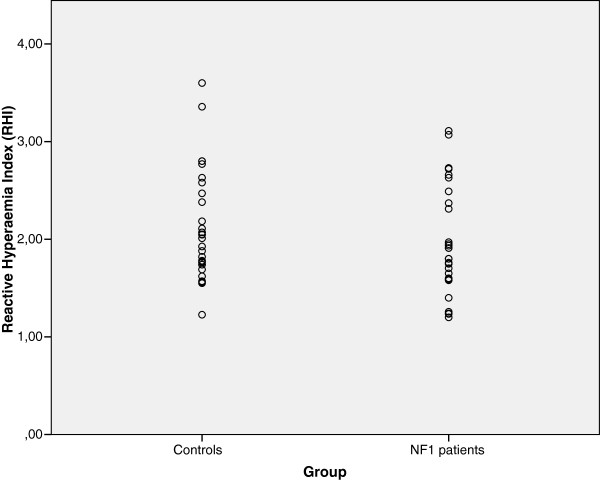
Scatter plot of reactive hyperemia index (RHI) values in both controls and patients with NF1.

The percentage of subjects with ED (RHI < 1.35) was 13.8% (4/29) in the NF1 group and 3.3% (1/30) in the control group (p = 0.153). The RHI medians and interquartile intervals were 1.8 (1.58-2.43) for the NF1 group and 2.02 (1.74 – 2.49) for the control group (p = 0.361). When the LnRHI means were compared using parametric tests, the difference was not statistically significant (p = 0.224). Figure 2 shows the distribution of all results in cases and controls as a scatter plot.

## Discussion

To our knowledge, this study is the first to evaluate endothelial function using PAT in NF1 patients. Contrary to expectations, these results suggest that the endothelial function of young adults with NF1 does not different from that of similar subjects without NF1.

Exacerbated vascular injury responses and chronic inflammation have been observed in murine models of NF1, and chronic inflammation in patients with NF1, suggesting that these factors could lead to ED [8]. Neither that study, nor studies on neurofibromin and endothelium in mice [6] and humans [7] tested endothelial function, although they did show that neurofibromin was expressed in endothelial cells [7] and that neurofibromin was important in cardiac embryogenesis in mouse models [6].

A death certificate analysis [4] reported a lower proportionate mortality ratio (PMR) for diseases of the circulatory system in individuals with than without NF1, especially for ischemic heart disease, which is closely related to atherosclerosis and endothelial dysfunction [15,16]. Moreover, in a similar study [3], although the PMR for vascular diseases in general was found to be higher in NF1 patients, the PMR for diseases of the arteries and arterioles, including atherosclerosis, was lower, a finding consistent with ours.

Our method of assessing endothelial function has several limitations. However, another method, the brachial artery reactivity test (BART), while being the most validated, requires extensive training and standardization. This has led to the development of alternative methods of evaluating endothelial function [17], including the PAT used here. We chose this method because it is simple, non-operator-dependent and commercially available, and because our research group has used it in patients with other diseases and has found its results reproducible [18].

Moreover, PAT and BART do not measure the same aspects of vascular biology. PAT measures the vasodilator response of the digital microvasculature to ischemia, whereas BART measures the response of the conduit arteries. The vascular bed and vessel size likely determine sensitivity to early damage from specific cardiovascular risk factors [19,20]. Therefore, the stage of disease progression may affect the ability of different methods to detect “abnormal” endothelial function. Nevertheless, both the PAT and BART methods correlate well with risk factors for CAD [15,19] and with coronary artery endothelial function [12,21] and both are able to predict future vascular events [16,22], although they correlate modestly, if at all, with each other [19]. Therefore, each method complements the other, providing additional information on endothelial function. BART likely provides more important information on individuals with existing atherosclerosis, whereas PAT may be an earlier indicator of CAD risk. Thus, PAT seemed more appropriate for profiling our study population.

Another concern about the PAT method was that the cutoff value was based on findings of a receiver operating characteristics (ROC) curve, which found that a cutoff value of 1.35 to diagnose patients with ED had a sensitivity of 80% and a specificity of 85% [12]. This cutoff value was based on patients referred for diagnostic percutaneous coronary angiography, a high-risk population with current or developing vascular disease. Thus, using the same cutoff value on a population of young, asymptomatic adults *without vascular disease risk factors*, as in our study, is questionable because it could change the predictive value of the test, especially if used for screening. We therefore used a combination of this cutoff value and LnRHI, which allowed for a comparison of mean values. Nevertheless, we found that the between group difference in mean LnRHI values was not statistically significant.

Only a few studies have used EndoPAT to non-invasively assess endothelial function in young subjects, including children, making it difficult to determine the expected prevalence of ED in the control group. One study tested the feasibility and reproducibility of EndoPAT in adolescents but did not indicate the percentage of subjects that presented with ED [23]. Similarly, a study that assessed ED in diabetic children [24] did not indicate how many of them had ED, as defined by the cutoff values. Since both studies compared mean and standard deviations of RHI values between groups, it was difficult to determine how many subjects in each group were below the cutoff value. Moreover, when establishing values in healthy control subjects, it should be remembered that the results of non-invasive endothelial function tests vary significantly in an individual on different days; however, it is not clear if this is due to physiologic oscillations in endothelial function or to the imprecision of the methods [25].

Several characteristics differed significantly between our NF1 and control groups, including height, body mass index of women and resting heart rate, but, after adjustment to account for these differences in the covariance analysis, none was found to affect RHI (data not shown).

One of the relative limitations of this study is its sample size; the observed difference in the prevalence of ED (10.5%) was lower than the difference expected (30%) when calculating sample size, thus reducing its *post hoc* power (30%). Although this sample was not powered to exclude small differences between groups, our findings suggest that ED is not clinically relevant in patients with NF1. This lack of difference in endothelial function suggests the need for further studies evaluating additional markers of ED, markers that are more sensitive and specific than PAT. In addition, older patients with NF1 should be included. These changes may result in a more precise determination of the function of neurofibromin in haploinsufficient endothelium.

Finally, understanding the natural history of NF1 vasculopathy requires prospective evaluations of the endothelial response of NF1 patients to risk factors of vascular disease. Our clinical observations suggested that atherosclerotic vascular disease risk factors, such as obesity, type 2 diabetes and hypertension, have lower prevalence in NF1 patients than in healthy controls, although this finding has not been confirmed. Thus, NF1 patients who die at a younger age due to vascular disease may do so from pathological mechanisms that may be different from those in the general population. Vascular disease in patients with NF1 may be caused by other factors, such as arterial fibromuscular dysplasia, rather than atherosclerotic disease [26]. Furthermore, both death certificate studies showed that patients with NF1 had a lower PMR for atherosclerosis-related conditions, including ischemic heart disease [4] and diseases of the arteries and arterioles [3], and a much lower PMR for atherosclerosis risk factors, such as diabetes [3,4] and hypertension [4].

These observations and our results are in agreement, that ED is not more prevalent in patients with NF1 than in the general population. Prospective assessment of vascular disease and endothelial function in patients with NF1 may provide greater understanding of this subject.

## Conclusion

The prevalence of endothelial dysfunction was no higher in NF1 patients than in healthy subjects.

## Competing interests

All the authors declare that they have no competing interests.

## Authors’ contributions

LOR collected the data, performed the statistical analysis and wrote the manuscript. LOCR conceived of the study, participated in its design and coordination, and helped draft the manuscript. LLC participated in the collection of data and revised the manuscript. NAR conceived of the study, participated in its design and coordination and helped draft the manuscript. ALPR performed the statistical analysis and helped draft the manuscript. All authors read and approved the final manuscript.

## Authors’ information

LOR has an MD and MSc, and this study was her master’s degree work at the Federal University of Minas Gerais.

LOCR has an MD and PhD, and is the Clinical Coordinator of the NF Outpatient Reference Centre of the state of Minas Gerais, Brazil.

LLC is a Medicine graduate student at the Federal University of Minas Gerais.

NAR has an MD and PhD, and is the General Coordinator of the NF Outpatient Reference Centre of the state of Minas Gerais, Brazil.

ALPR has an MD and PhD, and is the General Director of the *Hospital das Clínicas* of the Federal University of Minas Gerais.

## Pre-publication history

The pre-publication history for this paper can be accessed here:

http://www.biomedcentral.com/1471-2261/13/18/prepub
